# Radiological approach to metatarsalgia in current practice: an educational review

**DOI:** 10.1186/s13244-025-01945-3

**Published:** 2025-04-29

**Authors:** Océane Palka, Raphaël Guillin, Romain Lecigne, Damien Combes

**Affiliations:** 1 Department of Radiology, University Hospital of Rennes, Rennes, France; 2 Department of Radiology, University Hospital of Angers, Angers, France

**Keywords:** Forefoot, Metatarsalgia, Radiograph, Ultrasound, MRI

## Abstract

**Abstract:**

Metatarsalgia, characterized by forefoot pain, is frequent and is primarily due to foot static disorders. Initial evaluation with weight-bearing radiographs is essential, allowing precise analysis of the architecture of the foot. Ultrasound is useful for soft tissue and tendon examination and provides the best clinical correlation. Computed Tomography provides detailed bone assessment and is helpful for pre-operative planning. Magnetic Resonance Imaging is the gold standard modality, offering superior soft tissue contrast. The common causes of metatarsalgia include hallux pathologies (hallux valgus, hallux rigidus, and sesamoid issues), bursitis (intermetatarsal and subcapitellar), Morton’s neuroma, second ray syndrome, stress fractures, and systemic pathologies affecting the foot. Combining clinical and imaging data is crucial for accurate diagnosis and effective management of metatarsalgia. Post-traumatic causes of metatarsalgia are beyond the scope of this article and will not be described.

**Critical relevance statement:**

Metatarsalgia, the pain of the forefoot, necessitates accurate imaging for diagnosis and management. This review critically assesses imaging techniques and diagnostic approaches, aiming to enhance radiological practice and support effective therapeutic decision-making.

**Key Points:**

- Metatarsalgia commonly results from foot static disorders, requiring weight-bearing radiographs for assessment.
- MRI is often the gold standard examination, but ultrasound is complementary, allowing for a radioclinical approach with dynamic examinations.
- The radiologist is crucial in diagnosing metatarsalgia, providing essential imaging, and guiding treatment.

**Graphical Abstract:**

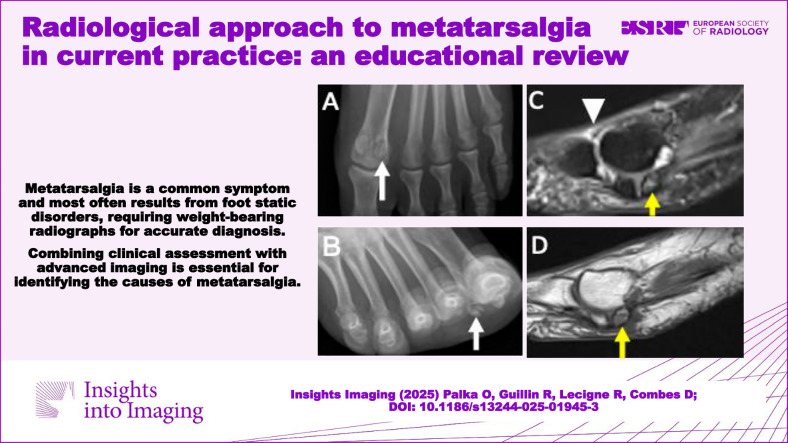

## Introduction

Metatarsalgia refers to pain in the forefoot, encompassing the metatarsal region, metatarsophalangeal joint (MTPJ), intermetatarsal space, and the soft tissues beneath the metatarsals, regardless of the underlying cause. The forefoot is commonly reported as the most frequent site of foot pain, affecting approximately 10% of the general population, with the prevalence rising in older adults due to age-related biomechanical changes [1].

The vast majority of metatarsalgia results from static disorders related to the architecture of the forefoot. Clinical history along with a thorough physical examination typically helps to narrow the differential diagnosis. However, imaging is essential for definitive diagnosis and appropriate management.

The aim of this educational review is to provide an overview of the primary imaging modalities and then to comprehensively detail the main causes of metatarsalgia.

## Imaging and measuring foot statics

### Main radiological views

The initial radiographic assessment of metatarsalgia typically begins with weight-bearing views, including standing dorso-plantar (anteroposterior) and lateral views of the foot and a non-weight-bearing medial oblique view.

Depending on the situation, a sesamoid view of the forefoot (Walter muller or Güntz incidence) may also be performed. This radiographic assessment is often sufficient to diagnose deformities and the main bone and joint pathologies [2].

### Foot statics measurements

**Fig. 1:**
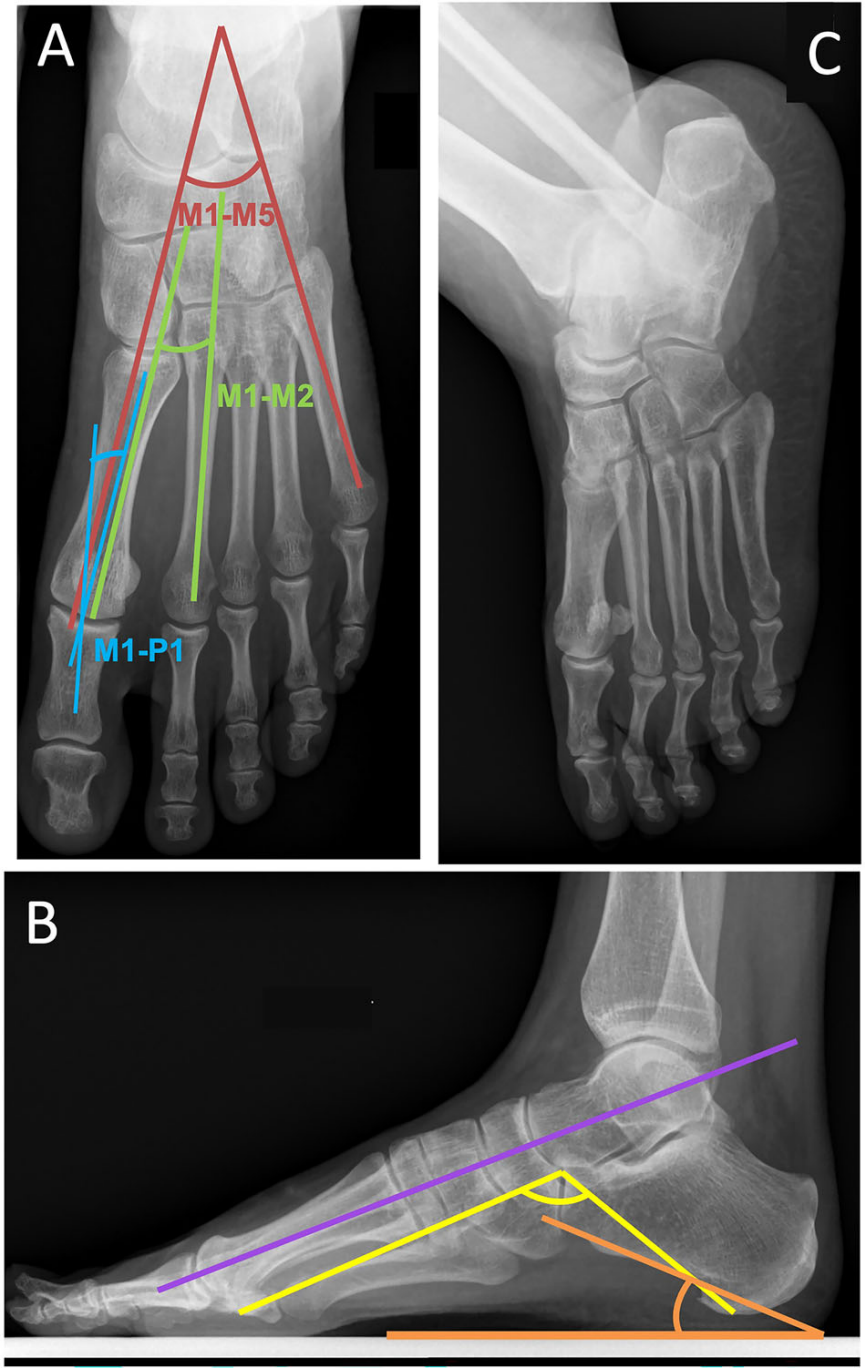
Main radiological views of the forefoot with measurements used in current practice. Weight-bearing dorso-plantar (**A**) and lateral (**B**) views and medial oblique view (**C**). In dorsoplantar view (**A**)—the metatarsophalangeal angle of the hallux: *N* < 15° (blue angle)—the metatarsus varus angle: *N* < 10° (around 5°) (green angle)—the angle of opening of the forefoot (width of the metatarsal fan): *N* between 15° and 20° (red angle). In profile view (**B**)—the Djian–Annonier’s angle (angle of the medial arch of the foot): *N* between 120° and 130° (yellow angle)—the talo metatarsal angle (Meary–Tomeno angle): normally forming a straight line (purple line). The slope of the calcaneus: *N* between 15° and 25° (orange angle)

A number of measurements are used on the radiographic assessment mainly to detect foot statics disorders (Fig. 1 and Table 1) [3–7].

**Table 1:** Main angles useful for studying foot statics on weight-bearing radiographs

Radiographic view	Angles	Definitions	Normal values	Pathologies
Lateral view	The Djian–Annonier’s angle (angle of the medial arch of the foot)	Angle between the calcaneal tubercle, the talonavicular joint (apex of the angle), and the medial sesamoid of the hallux	*N*: 120°–130°	> 130°: flat foot < 120°: direct hollow foot
	The Meary–Tomeno angle (talo metatarsal axis)	Longitudinal axis of the shell of the head of the talus and that of the first metatarsal	0°: forming a straight line	A break in this line: -With an angle with inferior apex: flat feet -With angle with superior: anterior hollow feet
	The slope of the calcaneus	angle of inclination between the line of the plantar surface of the calcaneus and the ground plane	*N*: 15°–25°	-Lowered in flat foot -Increased in the posterior hollow foot
	The slope of the metatarsals	Angle measured between the axis of each metatarsal and the plane of the ground	Decreases steadily from the first metatarsal (around 20°) to the fifth metatarsal (5°)	The angle of attack of the first metatarsal is reduced in flat foot and increased in hollow foot
Dorso plantar view	The metatarsophalangeal angle of the hallux	Angle between the axis of M1 and P1	*N* < 15°	Increased in Hallux Valgus
	The metatarsus varus angle	angle formed by the axes of M1 and M2	*N* < 10°	Increased in Hallux Valgus
	The metatarsophalangeal angle of the 5th ray	angle between the axis of M5 and that of P1 at the 5th radius	N between 8° and 10°,	> 10°: quintus varus

In the lateral view, the main angles are as follows:

- The Djian–Annonier’s angle (angle of the medial arch of the foot): angle between the calcaneal tubercle, the talonavicular joint (apex of the angle), and the medial sesamoid of the hallux (*N* between 120° and 130°). An angle > 130° defines a flat foot, < 120° a direct hollow foot [5];
- the talo metatarsal axis (Meary–Tomeno angle): longitudinal axis of the shell of the head of the talus and that of the first metatarsal, normally forming a straight line (0°). A break in this line with an angle with an inferior apex, is seen in flat feet and with an angle with a superior apex in anterior hollow feet [8];
- the slope of the calcaneus: angle of inclination between the line of the plantar surface of the calcaneus and the ground plane (*N* between 15° and 25°). It is lowered in the flat foot and increased in the posterior hollow foot;
- the slope of the metatarsals: the angle measured between the axis of each metatarsal and the plane of the ground. It normally decreases steadily from the first metatarsal (around 20°) to the fifth metatarsal (5°). The angle of attack of the first metatarsal is reduced in the flat foot and increased in the hollow foot;

The dorso-plantar view shows the metatarsal spread, and the respective lengths of the toes and metatarsals, which helps to identify any overloading caused by an excessively long metatarsal.

The digital formula classifies the feet into three types: Egyptian foot (if the size of the first ray > second ray), square foot (size of first ray = second ray), or Greek foot (size of second ray > first ray).

Various angles can be measured to study foot misalignments:

- the metatarsophalangeal angle of the hallux: angle between the axis of M1 and P1 (*N* < 15°).
- the metatarsus varus angle: the angle formed by the axes of M1 and M2. It is approximately 5° (*N* < 10°)
- the angle of opening of the forefoot (width of the metatarsal fan): the angle formed by the axis of M1 and that of M5 (N between 15° and 20°);
- the metatarsophalangeal angle of the 5th ray: angle between the axis of M5 and that of P1 at the 5th radius (N between 8° and 10°, beyond this, there is a quintus varus).

We can also analyze the position of the sesamoids measured according to the position of the medial sesamoid in relation to the lateral cortex of the head of M1 or the bisecting line of M1. There are three grades: Grade 0 (normal): the medial sesamoid remains medial to the bisecting line of M1; grade 1: < 50% overlap; grade 2: > 50% overlap; grade 3: the sesamoid lies beyond the bisecting line of M1 [9].

The medial oblique view is a complement to the dorso-plantar view of the foot, which allows the different metatarsals to be clearly separated. In our experience, it gives the better view of the joint spaces and helps detect arthropathy.

The sesamoid view (Walter-Müller Güntz view) is a tangential incidence on the sesamoids of the hallux. It enables a morphological study of the forefoot support zone, especially the sesamoids [6]. Their position is assessed in relation to the ventral rostrum of the head of M1.

### Other imaging techniques

Ultrasound (US) appears to be the second-line examination in metatarsalgia [2]. It is particularly useful for studying the soft tissues, bone surfaces, the integrity of forefoot tendons, and the plantar plate. The US can detect metatarsal fractures in appropriate clinical contexts even when conventional radiographs are negative [10]. This technique allows a dynamic study and is the most suitable for correlating the examination with the clinic. However, it is an operator-dependent technique. The evaluation should begin with an examination of the MTPJs. Dorsal exploration allows for the assessment of the cortical bone, cartilage of the metatarsal heads, synovium, and extensor tendons. The plantar examination focuses on the plantar plate, flexor tendons, and sesamoid bones. Dynamic maneuvers, including translation and ventral and dorsal flexion of the toe, can help detect subluxation. Additionally, the intermetatarsal spaces should be examined for signs of bursitis or interdigital nerve pathology, along with an assessment of the subcutaneous tissues.

Computed tomography (CT) is renowned for its effectiveness in assessing bone details and revealing cortical and trabecular pathologies, useful for the detection of subtle fractures or their characterization. It is essential for pre-operative planning. We can also mention the usefulness of dual-energy CT which has proved its worth in characterizing crystals especially uric acid crystals in gout [11].

Magnetic resonance imaging (MRI) is considered to be the reference examination on the basis of a well-founded clinical indication in order to adapt the segment analyzed and the appropriate protocol. It offers superior soft tissue contrast and detailed visualization of anatomical structures of the forefoot. Nonetheless, it is performed without weight-bearing, so radiographic assessment should be done systematically before proceeding with the MRI examination.

In our institution, the standard MRI protocol includes 3D T1 (Field of view (FOV) 180 mm; thickness 0.63 mm) and T2 weighted fat suppressed (FOV 187 mm; thickness 0.63 mm) isotropic sequences. In the absence of 3D, sagittal (FOV 160 mm; thickness 3.0 mm) and coronal T2 weighted fat suppressed (FOV 130 mm; thickness 2.5 mm), with coronal T1 (FOV 130 mm; thickness 2.5 mm) sequences should be performed [12].

Then, if there is a suspicion of metatarsal stress fracture you can add axial T1 and T2 FS in the plane of the metatarsals. If there is a suspicion of second-ray syndrome, a fine-centered sagittal DP FS sequence can be added.

Contrast injection is rarely required but can help differentiate Morton’s neuroma from a soft-tissue mass or assess suspected inflammatory rheumatism, bone tumors, or soft-tissue tumors. When contrast is used, post-gadolinium sagittal and coronal T1 Dixon sequences are preferred.

The use of new wrist/hand coils in MRI can significantly improve resolution if the forefoot fits within the coil. These coils also provide medial and lateral compression, mimicking Mulder’s maneuver, which is ideal for evaluating Morton’s neuromas. Additionally, they help reduce claw-like deformations during imaging.

Finally, some other imaging techniques should be mentioned including nuclear medicine techniques. Radionuclide imaging studies offer high sensitivity and provide an advantage in deducing functional information regarding bone turnover, rather than solely relying on the anatomical data [12, 13]. For example, scintigraphic assessment of the forefoot can be used as a diagnostic tool for identifying stress fractures, arthritis or infection [12, 14, 15].

## Metatarsalgia secondary to a disorder of foot statics

### Hallux pathologies

#### Hallux valgus

Hallux valgus is the most prevalent pathology of the forefoot. It is most often secondary but congenital in 25% of cases [16]. The risk factors are female gender, wearing narrow shoes with heels, an Egyptian-type foot, and a valgus flatfoot. It results from a triple deformity: lateral deviation of the proximal phalanx (P1) of the hallux in relation to the first metatarsal (M1), a medial deviation of the tip of M1 (metatarsus varus), pronation of the hallux on its axis.

**Fig. 2:**
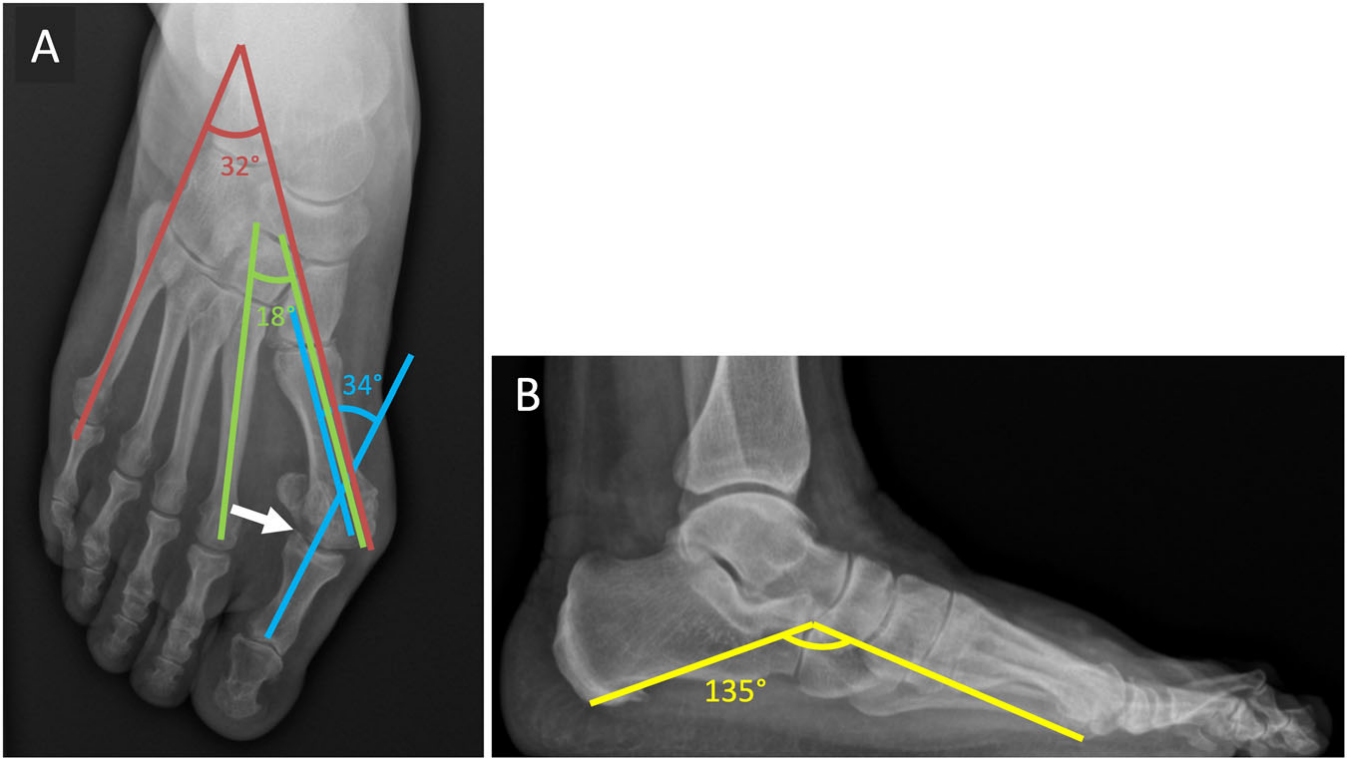
Hallux valgus of the right foot in a 63-year-old woman with a flat foot complicated by metatarsophalangeal osteoarthritis. Weight-bearing dorsoplantar (**A**) and lateral (**B**) radiographic views of the right foot. Forefoot opening angle (red angle): 32°; metatarsus varus angle (green angle): 18°; metatarsophalangeal angle of the first ray (blue angle): 34°; Toe formula: square foot; Djian–Annonier angle (yellow angle): 135° Degenerative arthropathy of the MTPJ (arrow), with moderate narrowing of the joint space, some subchondral bone changes of the condensing and microgeodic type, and a small amount of marginal osteophyte production

The imaging reference assessment is based on the standard weight-bearing radiographs of the foot (Fig. 2) [17, 18].

The dorsoplantar view of the foot reveals an enlargement of the metatarsophalangeal angle (angle M1–P1 > 15°) and a varus of M1 (angle M1–M2 > 10°).

The subluxation of the sesamoids is assessed both on the frontal and the sesamoid views. This last incidence may also reveal a hypoplasia of the crest in favor of the congenital origin of the deformity and osteoarthritis between sesamoid and metatarsal head.

The lateral view can reveal a flatfoot and dorsal dislocation of P1.

Moreover, radiographs allow for the evaluation of lateral osteoarthritis of the first MTPJ, microtraumatic changes to the medial aspect of the head of M1, and medial capital exostosis.

MRI and CT scan are not necessary for a simple hallux valgus. However, they may be indicated in order to assess the bone or adjacent soft tissues [19].

Medical treatment is limited to physical therapy, footwear advice, and orthotic use. Surgical treatment aims to realign the big toe, correct deformities, and relieve related pain. Conservative surgery typically involves metatarsal and phalangeal osteotomies, while joint fusion is reserved for severe or recurrent cases, especially in cases of advanced arthritic hallux valgus [20, 21].

#### Sesamoid pathologies

These bones are subject to numerous stresses, both static and dynamic. In this respect, they may be involved in traumatic or microtraumatic pathologies [22].

Sesamoiditis is a painful inflammatory condition resulting from repetitive injury to the plantar aspect of the forefoot. It corresponds to the edematous stage of sesamoid pathology. Diagnosis may be suspected in the US but is more easily made on MRI. US may reveal infiltration of the adjacent soft tissues, and hyperemia on Doppler imaging with elective pain on examination. Sesamoid bone marrow edema is visible through decreased or normal signal intensity on T1-weighted images and increased signal intensity on T2-weighted images (Fig. 4). Reactive soft-tissue abnormalities such as tendinitis, synovitis, and bursitis are typically associated findings [23, 24].

With persistent offending factors, a fracture can occur. Diagnosis must be made early in order to avoid necrosis. Radiographs can reveal fracture lines, separate fragments, periosteal reaction (old fracture). It can be suspected with US findings, (correlated with specific clinical features), revealing a cortical step-off but it is above all CT and MRI that enable it to be diagnosed early.

**Fig. 3:**
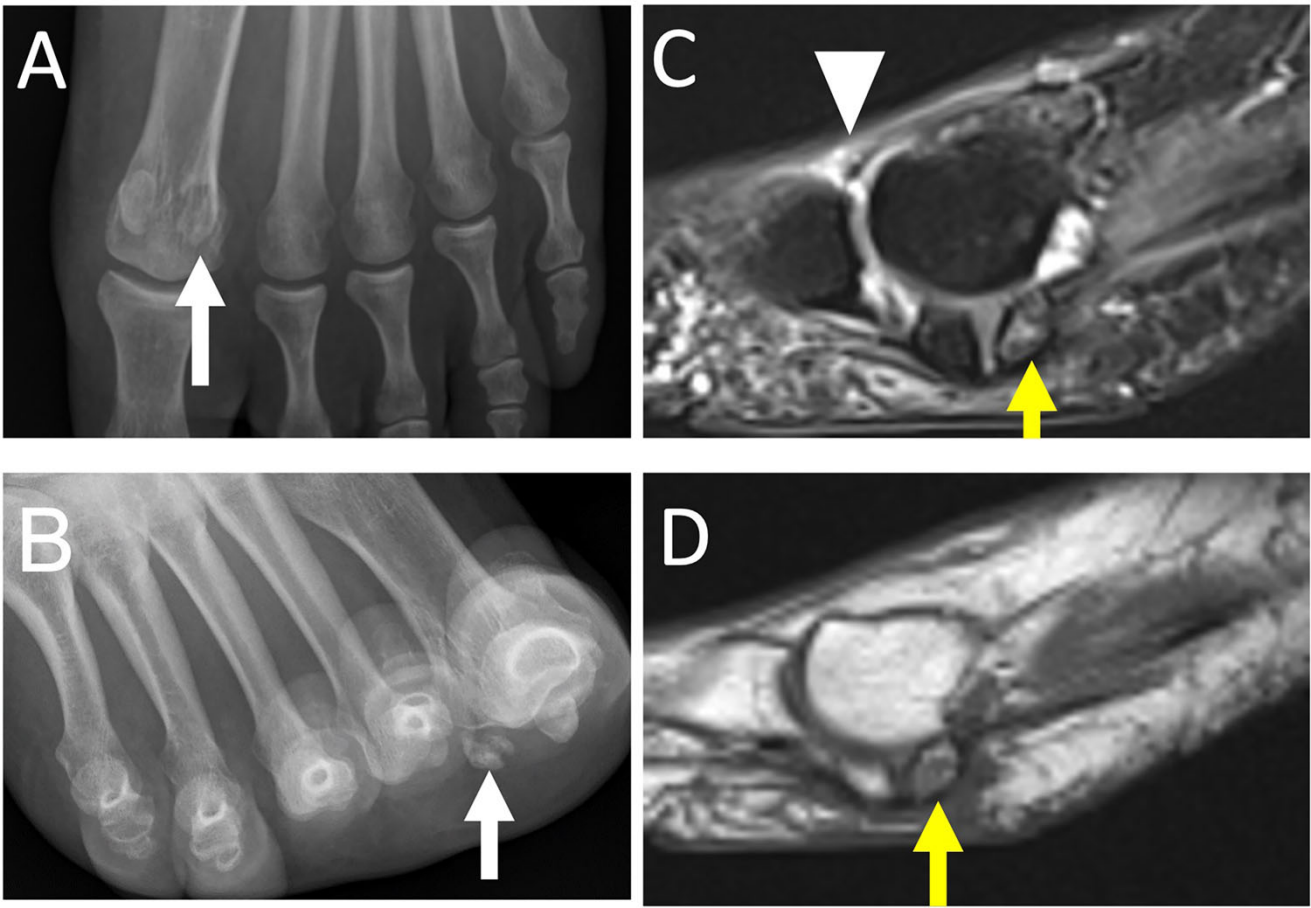
Chronic plantar pain of the first ray in the woman of 23 years old. Radiographs of the forefoot in weight-bearing dorso plantar (**A**) and sesamoid (**B**) views show a fragmented appearance of lateral sesamoid suggesting osteonecrosis (long white arrows). Unexplained right foot pain for several months in a 65-year-old woman. Sagittal MR images show increased signal intensity in T2-weighted (**C**) and decreased signal intensity in T1-weighted (**D**) of the posterior part of the bipartite medial sesamoid (short yellow arrows), associated with reactive synovitis and soft tissue inflammation (arrowhead), in favor of medial hallux sesamoiditis

Osteonecrosis is more common in women and affects both the medial and lateral sesamoids [22]. The US may reveal an irregular appearance of the affected sesamoid with infiltration of the soft tissues. A condensed or fragmented appearance can be seen on the radiographs or CT scan (Fig. 3). On T1-weighted MR images, the necrotic sesamoid appears hyposignal. On fat-suppressed T2-weighted images, the sesamoid is hypersignal during the edematous phase, whereas it is hyposignal in the necrotic phase [25, 26].

#### Hallux rigidus

This is metatarsophalangeal sesamoid osteoarthritis of the first ray, leading to pain and loss of dorsal flexion of the joint, without axial deviation.

It may be primary or secondary to a variety of causes: abnormal length of M1, osteochondritis dissecans of the head of M1, too posterior a position of the sesamoids, post-traumatic or post-surgical lesions [22, 27].

**Fig. 4:**
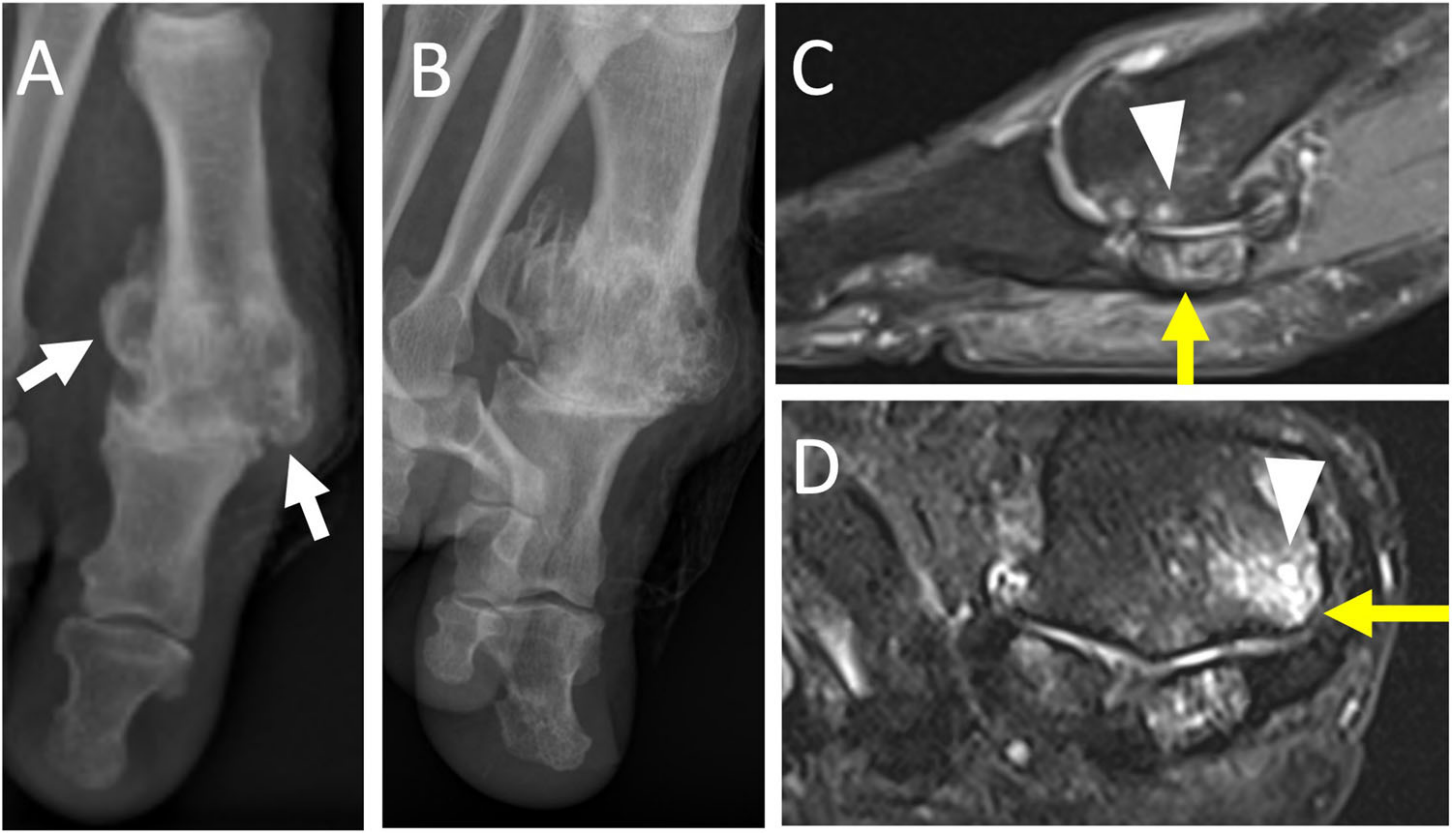
Hallux rigidus in a 66-year-old man. Radiographics in weight-bearing dorso plantar view (**A**) and medial oblique view (**B**) show severe metatarsophalangeal osteoarthritis of the first ray, with pinching of the joint, marginal osteophytosis (white arrows). Sagittal (**C**) and axial (**D**) MR images in T2-weighted fat-suppressed show joint effusion, microgeodic subchondral lesion (arrowheads) on the medial side of the base of the proximal phalanx and on the medial side of the head of the first metatarsal with associated metatarso sesamoid osteoarthritis with significant subchondral edema (yellow arrows)

Imaging shows severe arthropathy with narrowing of the first MTPJ, major pericapital osteophytosis, and in later stages ankylosis (Fig. 4) [28].

#### Joplin’s neuroma

Joplin’s neuroma corresponds to perineural fibrosis of the medial plantar digital nerve of the hallux, the terminal branch of the medial plantar nerve [29], due to microtrauma.

Patients present with pain and dysesthesia along the medial border of the hallux. The US shows a typical thickening of the nerve along the medial and plantar border of the hallux with a specific “trigger” zone [30]. MRI is also an effective diagnostic tool, but has the disadvantage of poor spatial resolution [29].

#### Knot of Henry intersection syndrome

The tendon of the flexor hallucis longus muscle crosses the dorsal surface of the tendon of the flexor digitorum longus muscle approximately 2 cm outside and below the navicular tuberosity. This crossing zone is called “Henry’s knot”. Acute or chronic hyperextension of the first MTPJ is then likely to cause tenosynovitis or longitudinal fissure of the flexor hallucis longus tendon at this level [31]. Diagnosis is mainly clinical, although US or MRI may also reveal signs of tenosynovitis or tendon fissure.

### Bursitis

Intermetatarsal bursitis is a fluid collection developed between two metatarsal heads, secondary to the inflammation of a physiological adventitious bursa [32]. This irritation may be mechanical, in most cases because of a forefoot static disorder, or secondary to inflammatory rheumatism. It is a prevalent cause of metatarsalgia, commonly linked with Morton’s neuroma and plantar plate tears. The expansion of the bursa must be more than 3 mm in transverse diameter but more importantly, it must exhibit a clinical correlation to be considered pathological [33].

**Fig. 5:**
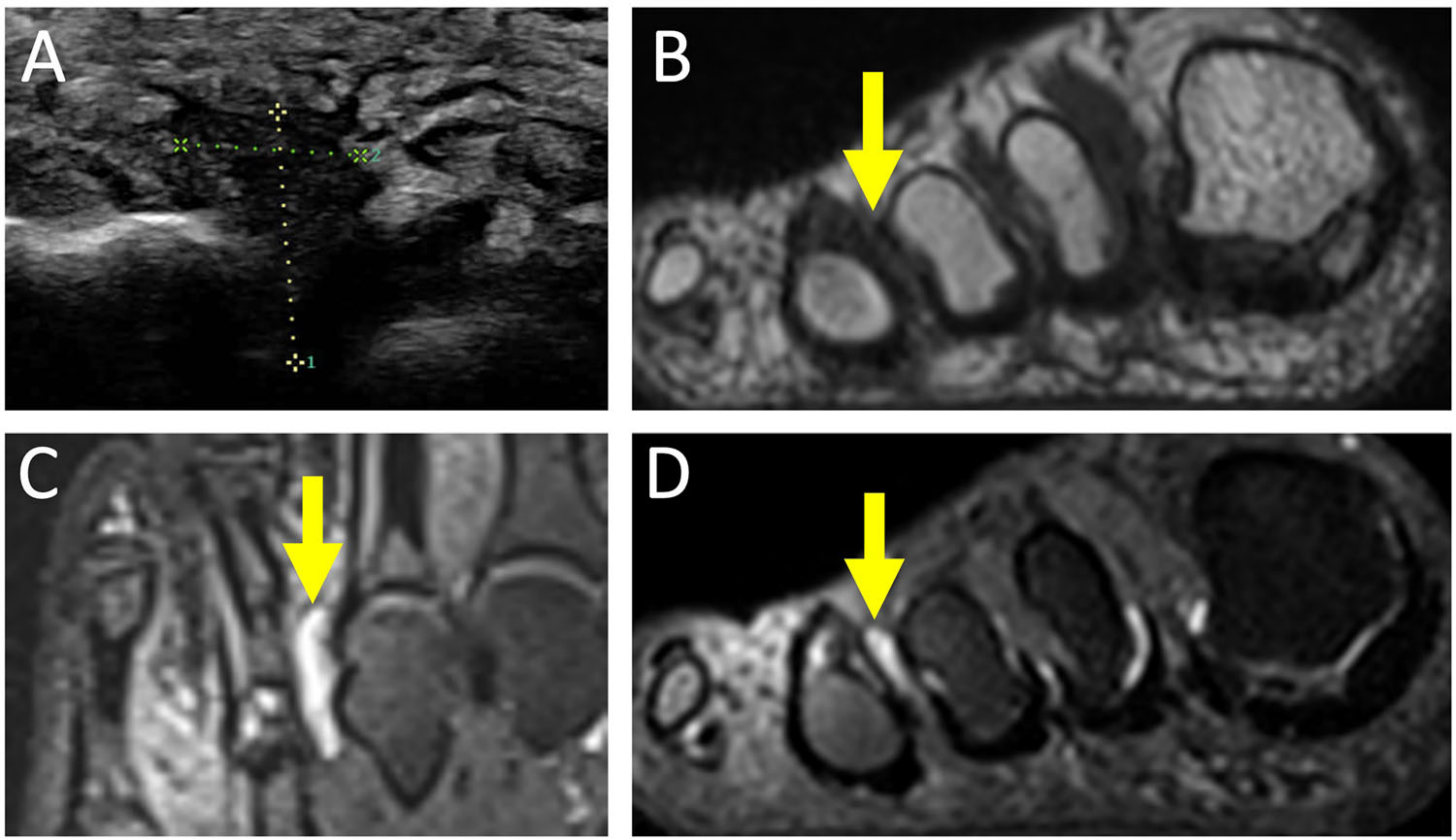
Intermetatarsal bursitis of the third space in a 48-year-old man presenting with right fourth toe pain aggravated by prolonged standing. US (**A**) shows a hypoechogenic mass of the third space which compresses in. MR images show a hypointense intermetatarsal mass in the T1-weighted short axis (**B**) and fluid hyperintense mass in the T2-weighted long and short axis (**C**, **D**) (arrows)

Due to its intermetatarsal location, it is poorly accessible in the US. It can reveal a thin-walled bursa distended with hypoechoic fluid collapsing under compression [2] and accompanied by peribursal hyperemia in acute bursitis. Chronic bursitis may exhibit a thickened bursal wall, and increased echogenic content. It can also mimic soft tissue masses or abscesses if marked synovial thickening is present. On MRI, the T1-weighted image detects mass syndrome, while the T2-weighted image aids in characterizing and distinguishing between fluid and tissue. Intermetatarsal bursitis appears hypointense on T1-weighted and fluid hyperintense on T2-weighted fat-suppressed images (Fig. 5). If gadolinium injection is performed, particularly in the case of an atypical appearance or location, peripheral enhancement may be observed.

**Fig. 6:**
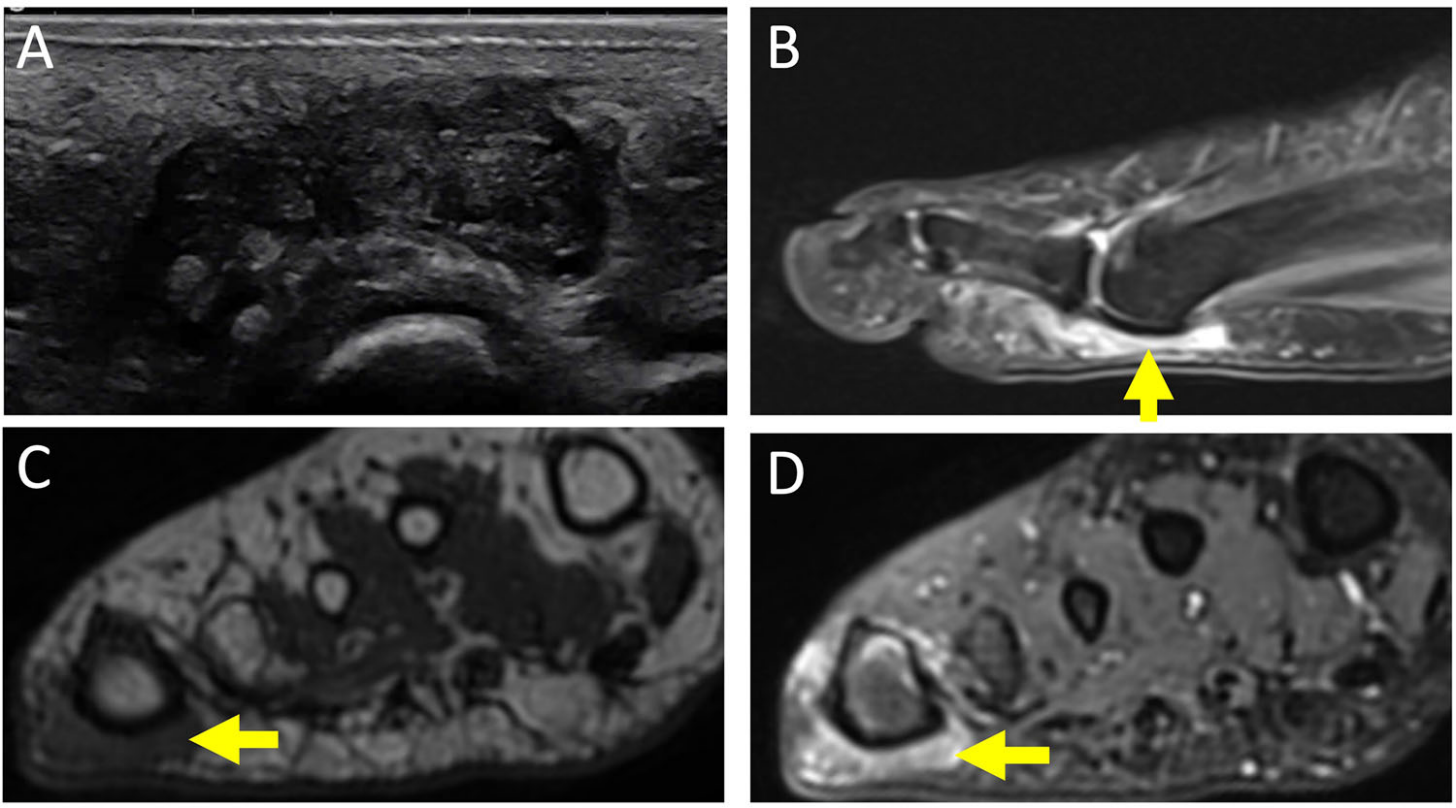
Submetatarsal bursitis in a 73-year-old woman with chronic left metatarsalgia. US (**A**) shows a hypoechoic edematous infiltration of the subcapito-metatarsal area of the fourth ray. MR images show hypointense in the T1-weighted short axis (**C**) and fluid hyperintense mass in the T2-weighted long and short axis (**B**–**D**) (arrows)

Subcapitellar metatarsal bursitis results from a fluid collection developed beneath a metatarsal head. The US is actually sufficient for diagnosis. In MRI, it exhibits similar signal characteristics to intercapitometatarsal bursitis but differs in location as it is subcapital (Fig.6) [2, 32].

### Morton’s neuroma

Morton’s neuroma corresponds to intra- and perineural fibrosis of the common plantar nerve at the level of its bifurcation. Nerve entrapment is considered the primary cause of Morton’s neuroma, in association with microtrauma and ischemia. Risk factors include increased weight bearing, high heels, and foot arch anomalies. It typically occurs in middle-aged women. Clinical manifestations include acute pain between the metatarsal heads, occasionally described as an electric shock, which is alleviated upon shoe removal but worsened or replicated by applying transverse pressure on the metatarsal heads.

Diagnosis is typically established through clinical history and physical examination. However, the complexity of cases and other diagnostic considerations may require the use of clinical imaging to validate the diagnosis.

**Fig. 7:**
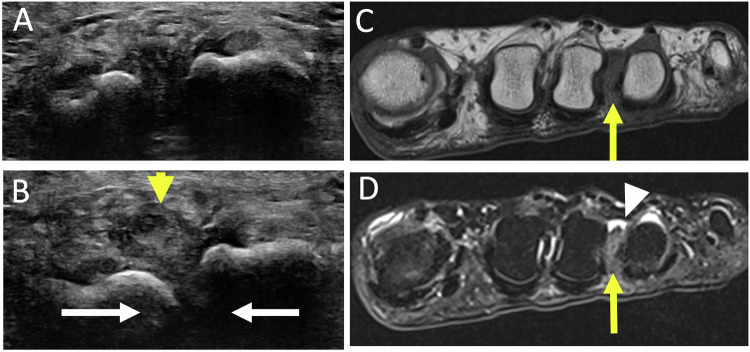
Morton’s neuroma in a 76-year-old woman presenting chronic left metatarsalgia with paresthesia of the fourth ray. US in short axis (**A**, **B**) shows hypoechoic mass located in the plantar intermetatarsal space, which dislocate (short arrow) during dynamic lateral compression (long white arrows) of the forefoot: Mulder’s sonographic sign MR images of the forefoot in short axis show oval mass in the third intermetatarsal space (long arrows), hypointense in T1-weighted image (**C**), hyperintense in T2-weighted image (**D**), overlain by a bursitis (arrowhead)

US is a highly effective modality for identifying Morton’s neuroma. It appears as a hypoechoic mass located in the plantar part of the second or third intermetatarsal space between two metatarsal heads, interrupting the hyperechoic inter-capito-metatarsal fatty column normally continuous with the subcutaneous fat [17, 34] (Fig. 7). The connection to the nerve can be clearly seen with the new US equipment, confirming the diagnosis without the need for an MRI scan [35]. The neuroma may dislocate towards the sole during transverse compression of the forefoot, with a visible and clinically perceptible protrusion: “Mulder’s sonographic sign” [36]. Unlike bursitis, the neuroma deforms but does not collapse during compression.

On MRI, Morton’s neuroma appears as a variable iso-hyperintense signal on T2-weighted images in the neurovascular bundle area, contrasting with the hyperintense fluid of the intermetatarsal bursa (Fig. 7). On T1-weighted images, it appears in the low-intensity signal. It often shows a characteristic dumbbell or ovoid shape on MRI, particularly in coronal views, due to the fusiform thickening of the nerve compressed between the metatarsal heads. Contrast after gadolinium injection varies in intensity but is always homogeneous, unlike bursitis where contrast is peripheral [17].

### Second ray syndrome (Fig. 8)

It corresponds to instability of the second MTPJ, arising from distension and subsequent rupture of the plantar plate, exacerbated by pre-existing degenerative lesions of the plantar plate [2]. This is favored by various conditions leading to an increased stress of the second MTPJ including a short first metatarsal, hallux valgus, an elongated second metatarsal, metatarsal verticalization (hollow foot, high heels), repetitive trauma to the forefoot, and iatrogenic factors (corticosteroid injections).

**Fig. 8:**
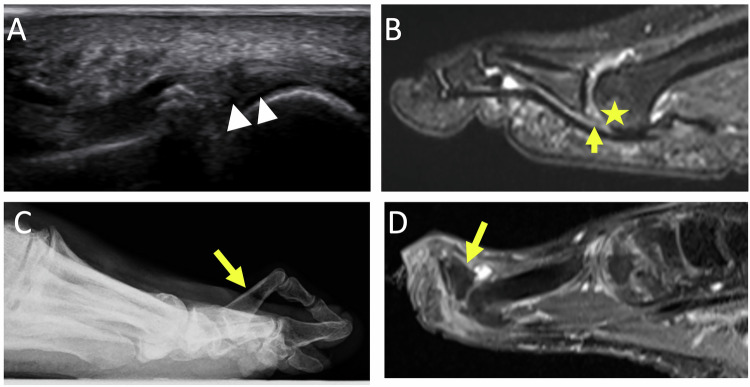
Second ray syndrome in a 34-year-old woman presenting with pain between the first and second intermetatarsal spaces for two months. US in sagittal plantar view (**A**) shows plantar plate irregularity (arrowheads), synovitis, and effusion of the 2nd MTP. Sagittal fat-suppressed T2-weighted MR image (**B**) shows rupture of the plantar plate (short arrow), with dorsal subluxation of P1, metatarsophalangeal synovitis with subchondral bone hypersignal of the head of the second metatarsal on its plantar side (star). Second ray syndrome in a 70-year-old man in terminal phase. Radiograph in lateral view (**C**) and sagittal MR image in T2-weighted (**D**) show dorsal dislocation of P1 (long arrows)

The progression of this syndrome typically unfolds in different phases [37]. In initial phase, instability of the second toe is associated with early damage to the plantar plate (simple painful phase). In state phase, subluxation or reversible dislocation of the second toe occurs due to elongation or even plantar plate rupture. In terminal phase, fixed dorsal dislocation of the base of P1 on the second metatarsal head results from plate rupture.

Radiographics are of little use in the early stages of the disease. They may show deviation of the proximal phalanx of the second ray in the early stage. Sagittal instability is best seen on a dorso-plantar view with pseudo-pinching of the joint space [2].

The US is the examination of choice. Dynamic dorsal dislocation maneuvers help to identify early instability. It may reveal a dorsal subluxation of P1, a lesion of the plantar plate, which may be thinned, partially or completely ruptured. Associated signs like synovitis and effusion of the second MTPJ and tenosynovitis of the flexor muscles of the second ray may be found. They can even precede plantar plate abnormalities [38, 39].

MRI can also be used to visualize signal abnormalities of the plantar plate (loss of homogeneity with areas of hyperintensity on T2-weighted images, thinning, rupture), dorsal subluxation of P1, arthropathy of the second MTPJ and tenosynovitis of the tendons of the flexor muscles [40]. However, it can be particularly challenging in MRI to detect early stages. Indeed, the presence of a physiologic small area of non-fluid hypersignal at the distal attachment of the plantar plate, visible in T2-weighted images may be confusing as it is difficult to distinguish this from a partial lesion [41].

## Stress fractures

Stress fractures result from an imbalance between bone strength and mechanical stress, constituting the primary bony cause of metatarsalgia [42]. Athletes, particularly runners and military recruits, are prone to fatigue fractures due to the excessive load on normal bone. It begins with bone stress injury, which refers to the bone’s inability to endure repetitive loading, leading to structural fatigue and localized pain and tenderness. This stress reaction can progress to a stress fracture and eventually result in a complete bone fracture. Insufficiency fractures occur due to typical loads on the bone that have been demineralized. They are more prevalent in women and elderly patients [43].

Metatarsal stress fractures frequently involve the middle and distal thirds of the diaphyses of the second and third rays. Much more rarely, this type of injury involves the cephalic subchondral bone plate [44, 45].

**Fig. 9:**
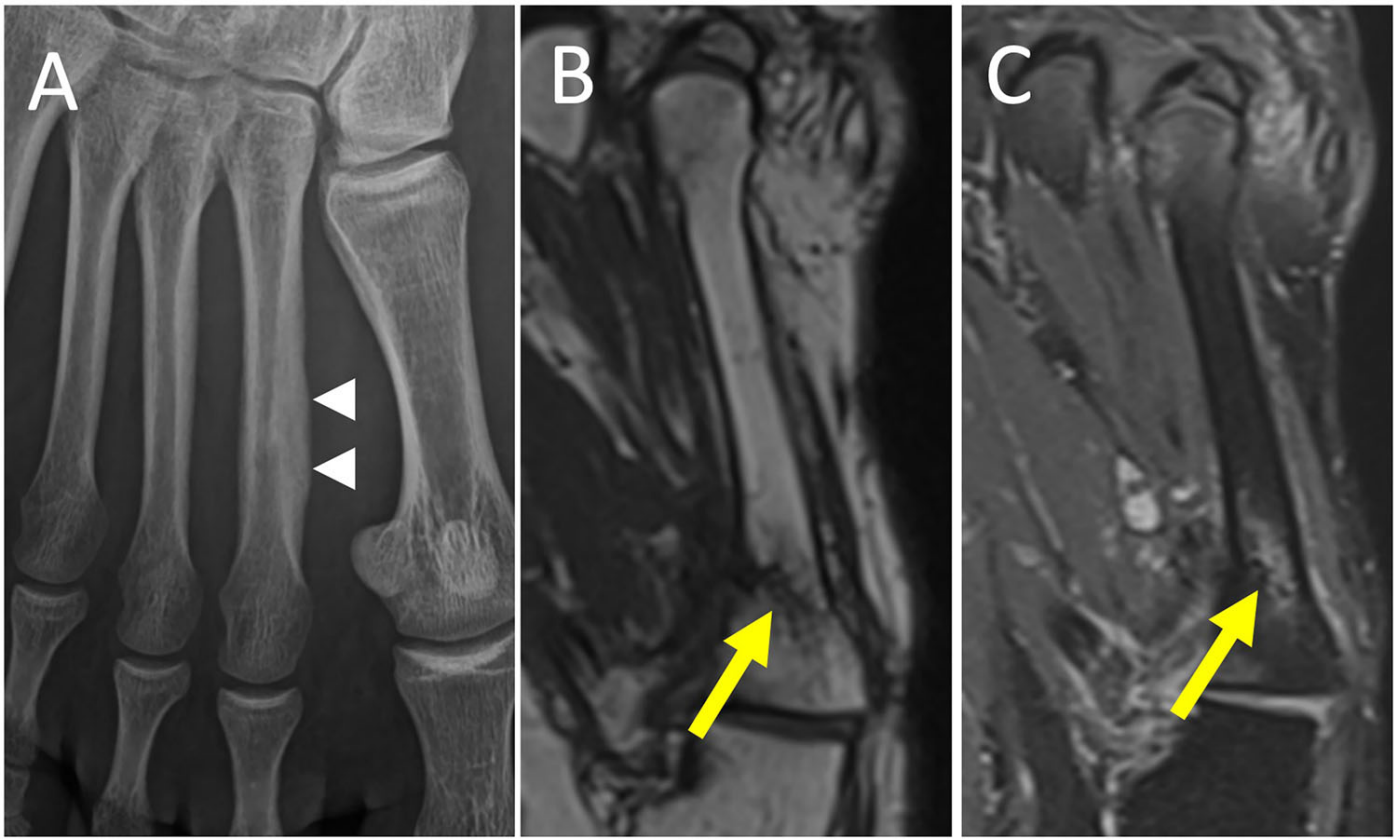
Stress fracture of the right second metatarsal in a 17-year-old patient. Radiograph in dorsoplantar view (**A**) taken retrospectively one month later, shows periosteal appositions of the second right metatarsal (arrowheads). A stress fracture in a 68-year-old man. MRI images show edematous intraosseous signal abnormalities in the proximal third of the shaft of the 4th metatarsal, surrounding a serpiginous hypo signal linear image (arrow) in T1 (**B**) and T2 (**C**): the appearance of a semi-recent stress fracture

Initial radiographs may yield negative results for up to two to three weeks. Detectable features include cortical fracture lines or the formation of callus (Fig. 9) [43].

US exhibits high and early sensitivity in detecting stress fractures, through periosteal thickening, cortical irregularities, and subcutaneous edema correlated with exquisite pain upon the passage of the probe [46].

MRI reveals a hypointense fracture line in T1-weighted images and bone marrow edema and surrounding reactive soft tissue edema in fluid-sensitive images [47] (Fig. 9).

## Osteochondrosis (Freiberg disease)

**Fig. 10:**
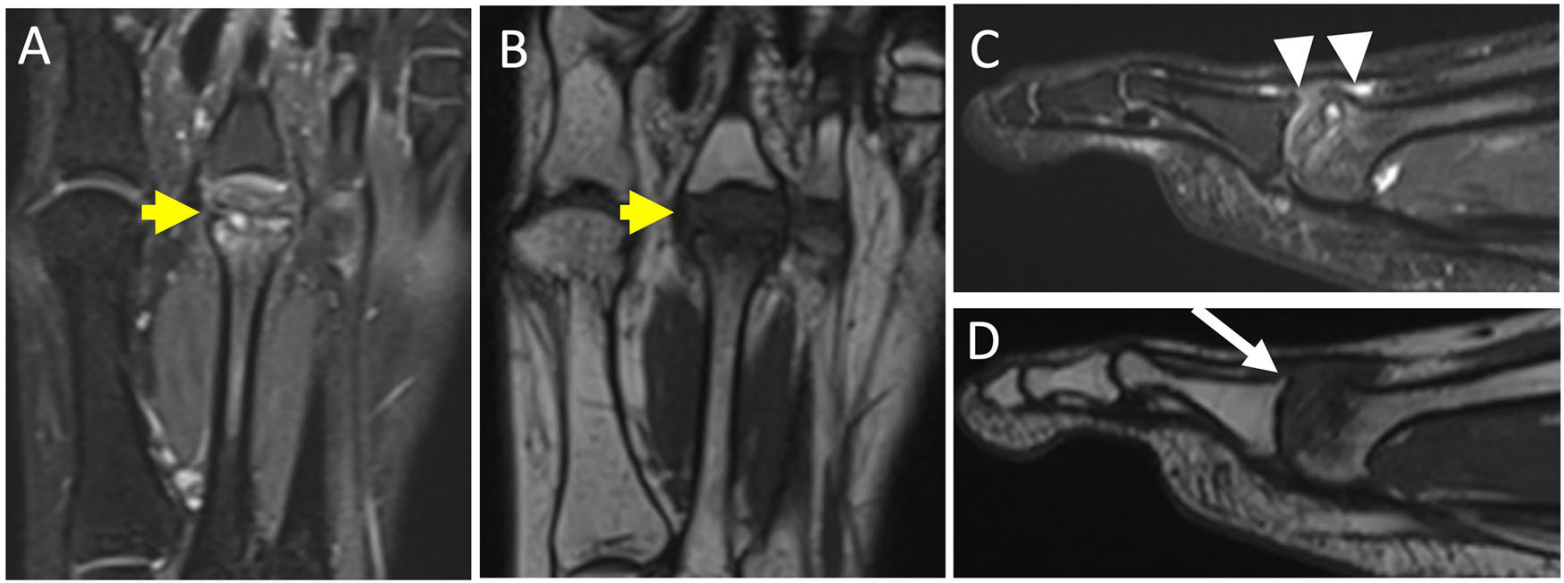
Freiberg’s disease of the second metatarsal of the left foot in the late degenerative stage in a 21-years-old woman. MR images in T2-weighted fat-suppressed (**A**–**C**) and T1-weighted (**B**–**D**) show deformity and hypertrophy of the head of the second metatarsal, edematous bone signal abnormalities (yellow short arrows) with geodic subchondral bone remodeling of the head, marginal osteophytosis of the base of proximal phalanx (long arrow), and intra-articular metatarsophalangeal effusion (arrowheads)

Freiberg infraction is a subchondral fracture primarily affecting the second metatarsal head in young patients and is a cause of mechanical metatarsalgia. It has a multifactorial etiology and the most commonly accepted hypothesis suggests that repeated microtraumas lead to a compression fracture of the subchondral bone, resulting in necrosis. This leads to the collapse and fragmentation of the epiphysis [43]. MRI findings precede radiographic evidence, revealing bone marrow edema, along with subtle subarticular and serpentine low signal linear abnormalities indicating the fracture line, accompanied by adjacent bone marrow edema [48] (Fig. 10).

Early radiographic signs include slight flattening and subchondral densification of the metatarsal head, occasional osseous transparency, and widened MTPJ space. As the condition progresses, characteristic abnormalities emerge, leading to a loss of the metatarsal head’s spherical shape. Diagnosis typically occurs late, revealing a shortened metatarsal diaphysis (due to premature growth plate fusion), hypertrophy, and deformation of the metatarsal head, with potential secondary MTPJ arthropathy [12].

## Systemic pathologies

### Septic arthritis

Arthritis may involve the MTP joints (Fig S2). Septic arthritis of the MTP joints is most commonly seen in diabetic patients and is caused by the spread of infection from an adjacent soft tissue or bone source [49]. Imaging findings include increased joint fluid and synovitis [50].

MRI can reveal edematous signal modifications with low signal intensity within the bone marrow on T1-weighted images, increased signal intensity on T2-weighted images, and contrast enhancement post-gadolinium. These findings are non-specific and may also be seen in degenerative, inflammatory, crystal arthropathy, or systemic diseases, especially if multiple joints are involved [42]. Arthritis can progress to osteomyelitis, in which case MRI may reveal marrow replacement (very low T1 signal, high T2 signal, and contrast enhancement after gadolinium), along with potential periosteal reaction, cortical disruption, or even abscess formation [51].

### Crystal arthropathy

**Fig. 11:**
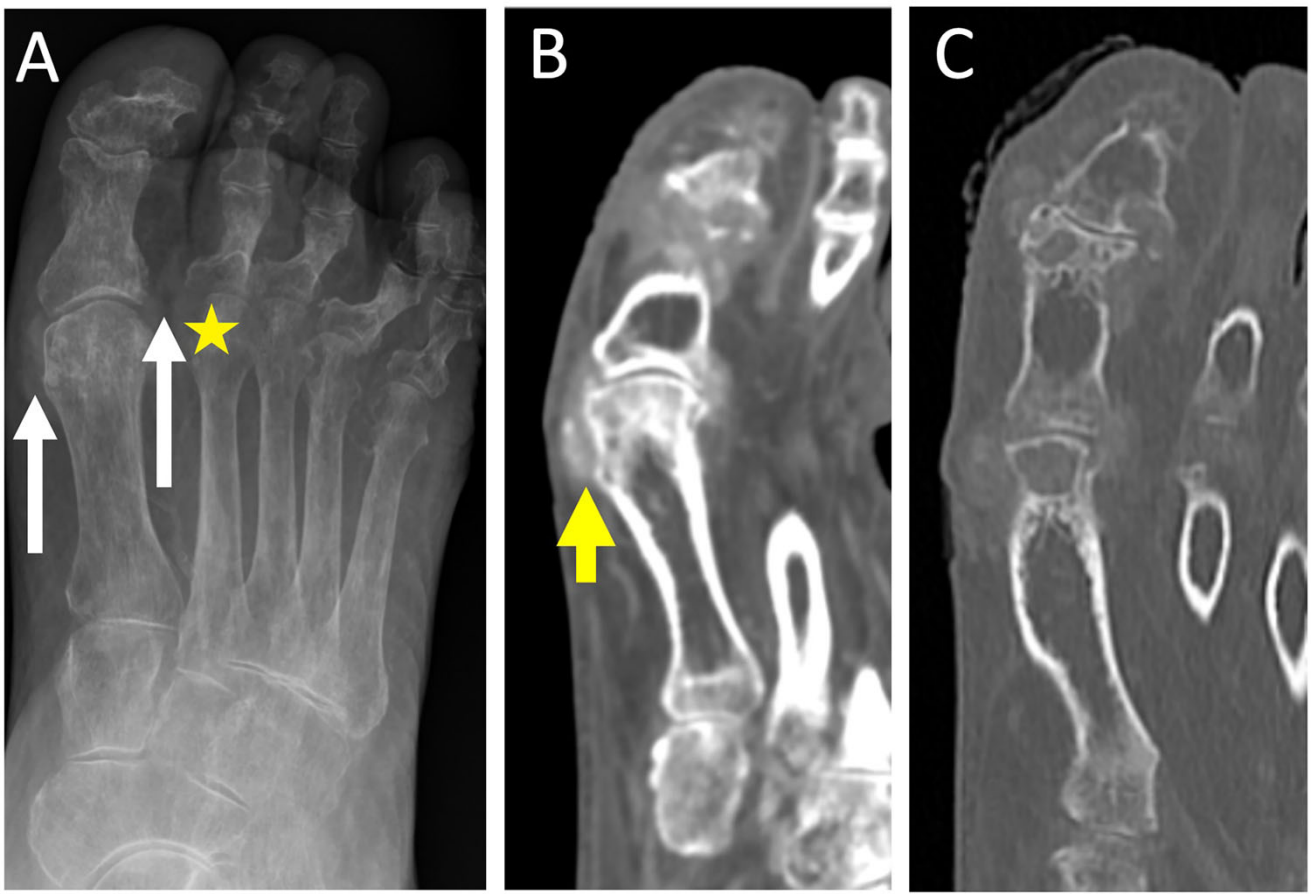
Chronic tophaceous gout in an 86-year-old man. Radiograph in dorso-plantar view (**A**) shows gouty tophus of the periarticular soft tissues opposite the MTPJ of the first and second rays (long arrows), bone erosion of the medial edge of the head of the second metatarsal (star), distal interphalangeal arthropathy. The MTPJ line spacing of the first ray is relatively intact compared to the extent of the erosions. CT (**B**, **C**) shows soft tissue thickening over the MTPJ of the hallux, containing a few discrete calcifications suggestive of gouty tophus (short arrow)

Crystals can accumulate in and around various structures in the forefoot, including joints, bones, tendons, bursae, and periarticular tissue. Among the main ones that can affect the foot, gout typically affects the first MTPJ. It is caused by sodium urate crystal deposition in joints and periarticular soft tissues. Acute gout is characterized by nonspecific imaging features such as joint effusion and synovial thickening. Tophaceous gout, the chronic form, can lead to bone erosions and present as soft-tissue masses at intra-articular or periarticular sites. In the US, tophi are visualized as hyperechoic soft tissue masses within subcutaneous tissues and around joints, often featuring a central hypoechoic zone [12]. On CT, tophi appear as soft tissue masses with a density of 160–170 HU and may exhibit calcifications [52] (Fig. 11). MRI revealed erosions and tophi appear iso-intense signal to muscle on T1-weighted images with variable enhancement following gadolinium administration [12, 53].

Among common microcrystalline conditions, calcium pyrophosphate deposition disease can affect the foot. Involvement of the first MTPJ may clinically and radiographically mimic gout [54]; however, the presence of calcifications and the absence of bone erosions typically aid in making the diagnosis.

Dual-energy CT has been shown to be a sensitive, noninvasive, and reproducible method for identifying uric acid deposits in joints and periarticular soft tissues in patients suspected of having gout, offering a diagnostic imaging option for patients who do not undergo joint aspiration [11]. It allows differentiation between uric acid crystals and pyrophosphate crystals.

### Rheumatoid arthritis

Rheumatoid arthritis commonly affects the MTP joints, frequently earlier than hands and wrists [12, 55]. Radiographs show early signs like soft tissue swelling and juxtaarticular osteopenia, and advanced manifestations such as marginal erosions, joint space narrowing, and malalignment (Fig. S1). US is increasingly employed for early disease assessment, efficiently detecting joint effusion, synovitis, subarticular erosions in the MTPJ. It is also useful for evaluating soft tissue changes, including tenosynovitis, tendinopathy, and rheumatoid nodules [56].

MRI is a sensitive modality to evaluate inflammatory and structural lesions in rheumatoid patients (joint effusions, synovial thickening, bursitis, and bone erosions). It can also reveal bone marrow edema, serving as an indicator of active inflammation [55, 57].

### Tendon disorders

Tendon disorders can affect the forefoot, and include tendinosis, tenosynovitis, tears (partial and complete), subluxation, dislocations, and occasionally, tendon entrapments [58]. Tendinosis means degeneration. On MR imaging, it is visible as a thickening with increased signal on T2-weighted images (Fig. S3). Tenosynovitis involves inflammation of the tendon sheath. It may particularly affect the flexor hallucis longus tendon between the sesamoid bones (subject to repetitive impact), and under the base of the first metatarsal bone, where the flexor digitorum longus crosses under the flexor hallucis longus [59]. The US reveals changes in tendon thickness and varying degrees of hypoechogenicity. MRI displays enlargement and signal alterations in affected tendons, with high signal intensity extending to peritendinous soft tissues. Tendon tears are hypoechoic in the US and show a fluid signal on T2-weighted images. Complete ruptures are often accompanied by retraction.

### Plantar venous thrombosis

**Fig. 12:**
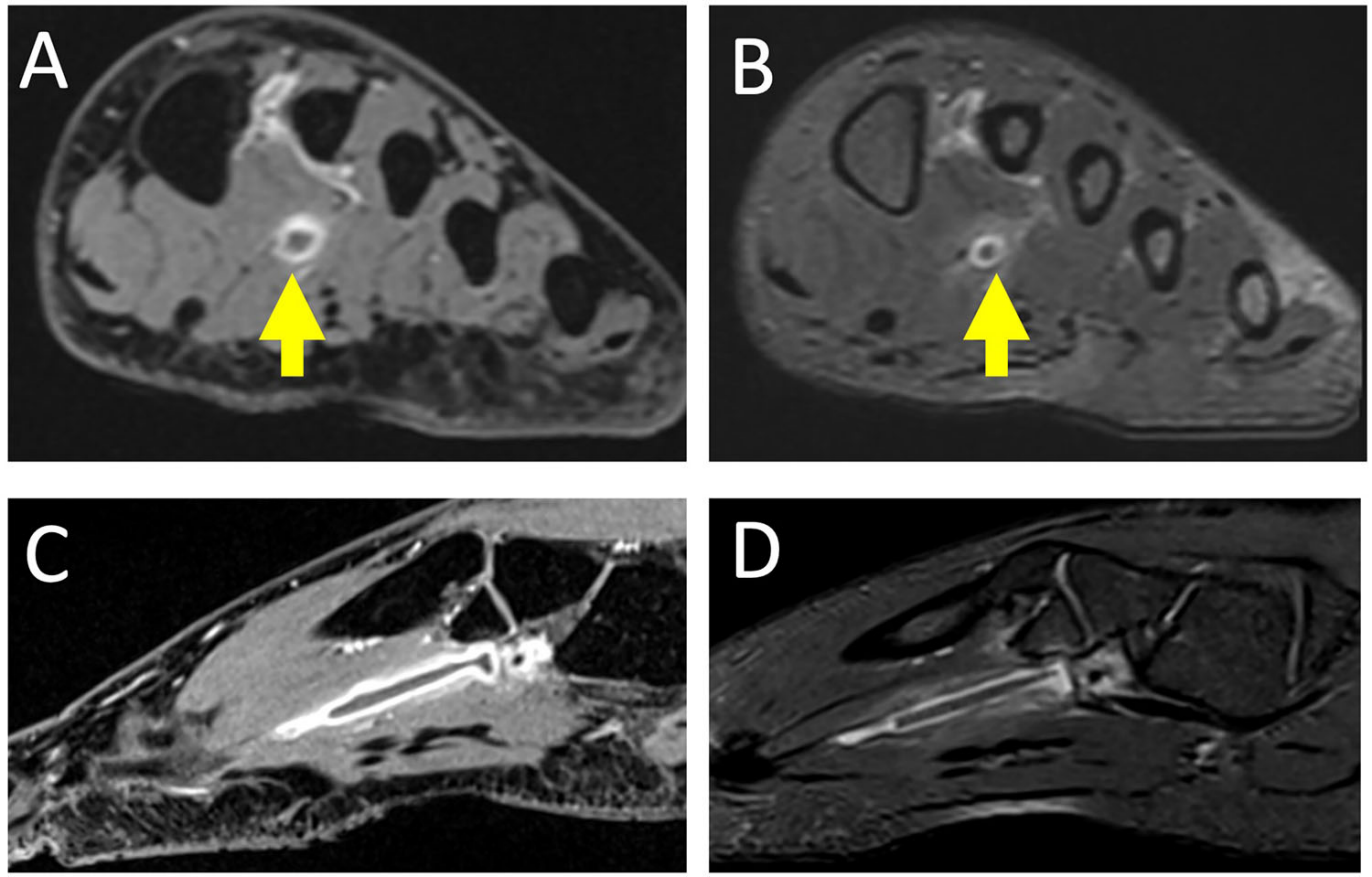
Plantar medial venous thrombosis in a 52-year-old woman presenting with pain in the foot for five days. MR images of the forefoot in T1 weighted fat-suppressed post gadolinium short and sagittal axis (**A**–**C**) and T2-weighted fat-suppressed (**B**–**D**) show acute thrombosis with venous defect in the medial plantar vein (arrows) with perivascular edema

Plantar vein thrombosis impacts the deep plantar veins, presenting with non-specific localized pain in the plantar foot, swelling, and a sensation of fullness, it is often underdiagnosed. The lateral plantar vein is affected in most of the cases [60]. US is the method of choice for diagnosis, it can show loss of compressibility and venous enlargement, intraluminal content, and perivascular edema with local tenderness [61]. MRI shows perivascular edema and enhancement, intraluminal signal change and venous enlargement, presence of collateral veins, and venous filling defects in post-gadolinium injection images (Fig. 12) [61, 62].

## Conclusion

Metatarsalgia is common and usually results from foot static disorders. Weight-bearing radiographs are essential and often sufficient for etiological diagnosis. However, the US with its advantage of enabling dynamic maneuvers and good clinical correlation, along with MRI, which allows for the most precise study of the anatomical structures of the forefoot, collectively provide further diagnostic precision and subsequently guide management.

Some pathologies, such as stress fractures, should not be missed because of the need for rapid offloading. Common pathologies such as bursitis, Morton’s neuroma and second ray disorders are often the result of a chain reaction of static disorders. As far as treatment is concerned, in addition to podiatric treatment with foot orthoses, interventional radiology techniques and in particular corticosteroid infiltrations offer promising prospects before considering surgical intervention.

## Supplementary information

ELECTRONIC SUPPLEMENTARY MATERIAL

## Data Availability

All the data in this manuscript are taken from a review of the scientific literature.

## Abbreviations

CT: Computed tomography
MRI: Magnetic resonance imaging
MTPJ: Metatarsophalangeal Joint
US: Ultrasound
