# Supplementary material for: Radiological approach to metatarsalgia in current practice: an educational review
Source: Insights Imaging. 2025 Apr 29;16:94. doi: 10.1186/s13244-025-01945-3 (PMC12041408; doi:10.1186/s13244-025-01945-3)

Radiological approach to metatarsalgia in current practice: an educational review.

ELECTRONIC SUPPLEMENTARY MATERIAL

Fig. S1 Destructive rheumatoid arthritis in a 60-year-old man.

Radiographs in dorso plantar (A) and medial oblique (B) views show juxtaarticular osteopenia (arrowheads), marginal erosions (stars), joint space narrowing, and malalignment.

US (C) detects joint effusion (arrow), synovitis, subarticular erosions in the metatarsophalangeal joint

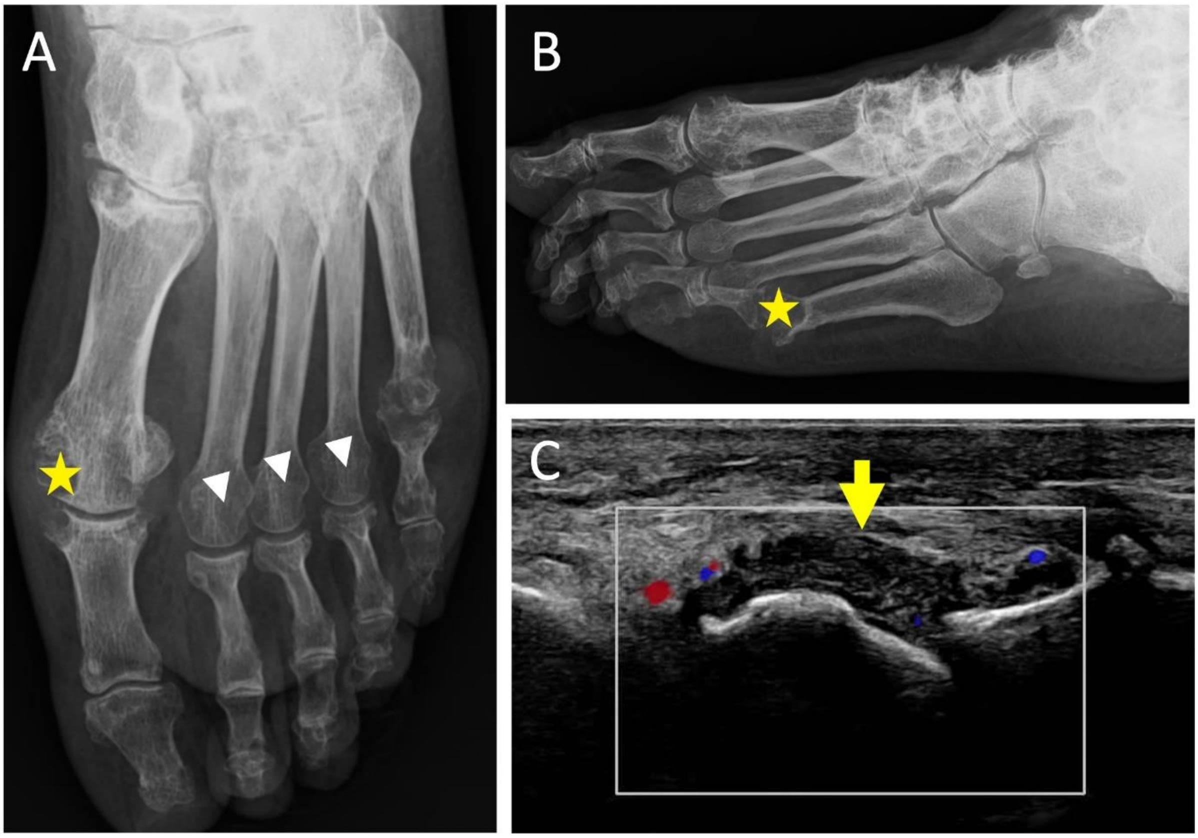

Fig S2. Septic arthritis of the first metatarsophalangeal joint.

MR images of the forefoot in sagittal T2 weighted fat-suppressed (A) and T1-weighted (B) show metatarsophalangeal joint pinch of the first ray, with joint fluid effusion (arrows) and soft tissue infiltration. Small upper para-articular collection (arrowhead). Reactive oedematous bone marrow signal change (star) and marked T1 hyposignal compatible with associated osteitis.

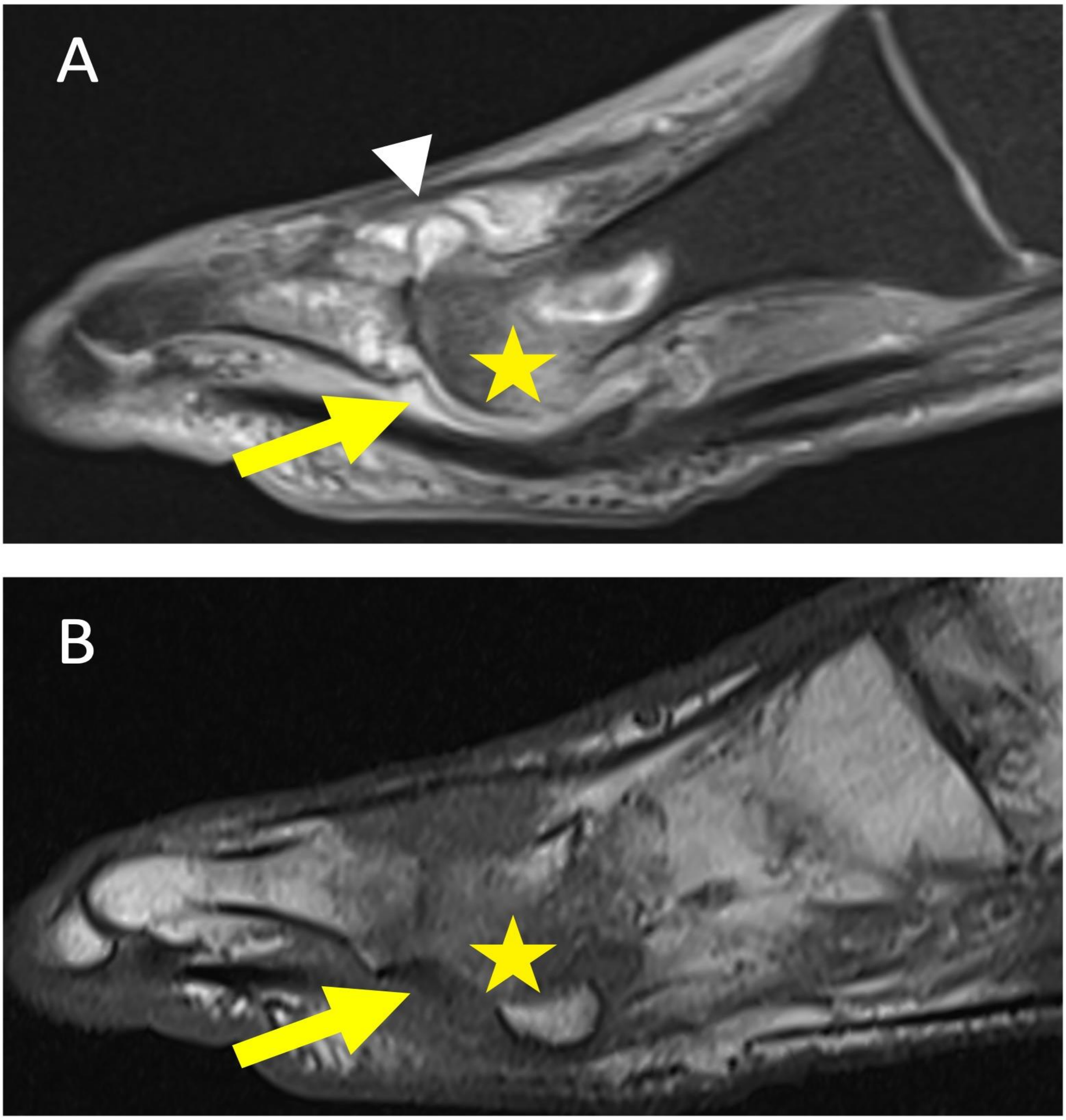

Fig S3. Tenosynovitis of the long flexor tendon of the second ray

MR images of the forefoot in T2 weighted fat-suppressed (A-C) and coronal T1-weighted (D) show an elevated signal from the flexor pollicis longus tendon associated with a peritendinous fluid effusion (arrows) and infiltration of the adjacent soft tissues.

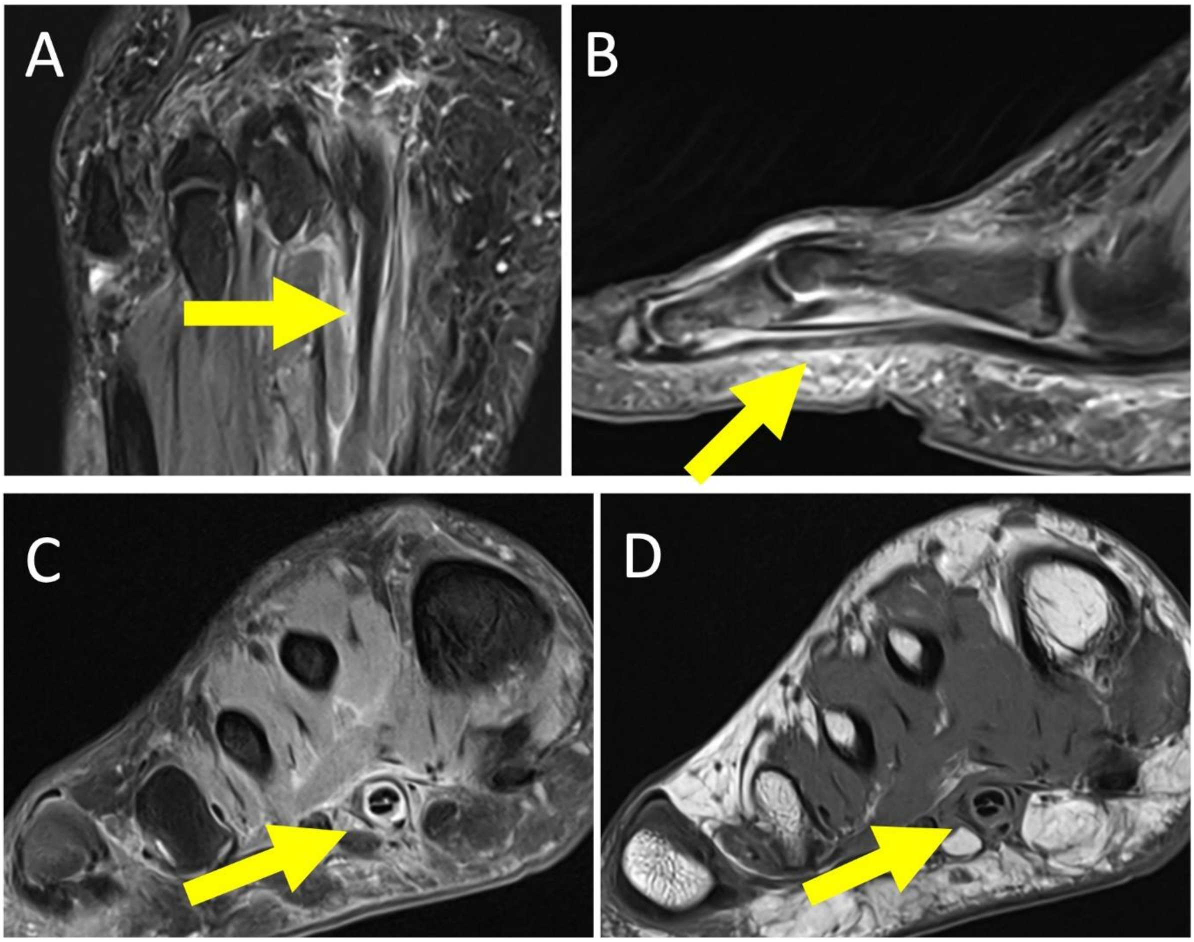

Supplement: Supplementary file 1 — ELECTRONIC SUPPLEMENTARY MATERIAL [file 13244_2025_1945_MOESM1_ESM.pdf]
